# A Heterozygous ZMPSTE24 Mutation Associated with Severe Metabolic Syndrome, Ectopic Fat Accumulation, and Dilated Cardiomyopathy

**DOI:** 10.3390/cells5020021

**Published:** 2016-04-25

**Authors:** Damien Galant, Bénédicte Gaborit, Camille Desgrouas, Ines Abdesselam, Monique Bernard, Nicolas Levy, Françoise Merono, Catherine Coirault, Patrice Roll, Arnaud Lagarde, Nathalie Bonello-Palot, Patrice Bourgeois, Anne Dutour, Catherine Badens

**Affiliations:** 1 Inserm UMR_S U910, Faculté de Médecine, Aix-Marseille Université, Marseille 13385, France; galant.damien@gmail.com (D.G.); camille.desgrouas@univ-amu.fr (C.D.); nicolas.levy@univ-amu.fr (N.L.); francoise.merono@univ-amu.fr (F.M.); patrice.roll@univ-amu.fr (P.R.); arnaud.lagarde@ap-hm.fr (A.L.); Nathalie.BONELLO@ap-hm.fr (N.B.-P.); Patrice.BOURGEOIS@ap-hm.fr (P.B.); 2 Inserm U1062/Inra1260, Aix-Marseille Université, Marseille 13385, France; benedicte.gaborit@ap-hm.fr (B.G.); inesabdesselam@hotmail.com (I.A.); anne.dutour@ap-hm.fr (A.D.); 3 APHM, Endocrinology, Metabolic Diseases and Nutrition, Marseille 13385, France; 4 Laboratoire de Chimie Analytique, Faculté de Pharmacie, Aix-Marseille Université, Marseille 13385, France; 5 CNRS, CRMBM UMR 7339, Aix-Marseille Université, Marseille 13385, France; monique.bernard@univ-amu.fr; 6 APHM, CHU de la Timone, Laboratoire de Génétique Moléculaire, Marseille 13385, France; 7 Institut de Myologie, UMR_S U974 INSERM-UPMC-CNRS-AIM, Paris 75013, France; c.coirault@institut-myologie.org; 8 APHM, CHU de la Timone, Laboratoire de Biologie Cellulaire, Marseille 13385, France

**Keywords:** metabolic syndrome, cardiomyopathy, ZMPSTE24, premature senescence, nuclear anomalies, laminopathy

## Abstract

*ZMPSTE24* encodes the only metalloprotease, which transforms prelamin into mature lamin A. Up to now, mutations in ZMPSTE24 have been linked to Restrictive Dermopathy (RD), Progeria or Mandibulo-Acral Dysplasia (MAD). We report here the phenotype of a patient referred for severe metabolic syndrome and cardiomyopathy, carrying a mutation in *ZMPSTE24*. The patient presented with a partial lipodystrophic syndrome associating hypertriglyceridemia, early onset type 2 diabetes, and android obesity with truncal and abdominal fat accumulation but without subcutaneous lipoatrophy. Other clinical features included acanthosis nigricans, liver steatosis, dilated cardiomyopathy, and high myocardial and hepatic triglycerides content. Mutated fibroblasts from the patient showed increased nuclear shape abnormalities and premature senescence as demonstrated by a decreased Population Doubling Level, an increased beta-galactosidase activity and a decreased BrdU incorporation rate. Reduced prelamin A expression by siRNA targeted toward *LMNA* transcripts resulted in decreased nuclear anomalies. We show here that a central obesity without subcutaneous lipoatrophy is associated with a laminopathy due to a heterozygous missense mutation in ZMPSTE24. Given the high prevalence of metabolic syndrome and android obesity in the general population, and in the absence of familial study, the causative link between mutation and phenotype cannot be formally established. Nevertheless, altered *lamina* architecture observed in mutated fibroblasts are responsible for premature cellular senescence and could contribute to the phenotype observed in this patient.

## 1. Introduction

Nuclear envelope defects, and more specifically defects of the meshwork named *lamina,* which underlie the nuclear envelope (NE), result in various rare diseases with premature aging features. Lamins (A and B-type lamins) are the main constituents of the *lamina*, which provide shape and rigidity to the nucleus and play a critical role in gene expression. To be fully functional, the lamin A precursor protein named prelamin A, undergoes several steps of post-translational maturation ending in the cleavage of its farnesylated tail and resulting in mature lamin A. ZMPSTE24, a zinc metalloprotease, is the only enzyme able to perform this cleavage and so far, prelamin A is the only mammalian substrate identified for ZMPSTE24 [1]. Progeria, one of the most severe diseases with premature aging, is most commonly caused by a dominant mutation (c. 1824C > T) in *LMNA*, the gene encoding A-type lamins [2,3]. This mutation leads to an internal deletion of 50 amino acids and removes the ZMPSTE24 cleavage site within prelamin A. Consequently, the resulting mutant prelamin A (called progerin) cannot be cleaved and accumulates in cells, exerting a toxic effect by NE perturbation [4]. Familial Partial Lipodystrophy syndromes (FPL) also result from heterozygous mutations in lamin A, often producing maturation defects and prelamin A accumulation, although less drastic than in Progeria.

Because of the critical role of ZMPSTE24 during prelamin A maturation, null mutations in *ZMPSTE24* also result in prelamin A accumulation and produce diseases with premature ageing [5,6]. Prelamin A that accumulates because of decreased ZMPSTE24 activity, permanently retains its farnesylated tail, similarly to progerin, and is responsible for the same nuclear defects. The amount of prelamin A which remains uncleaved varies according to the type of ZMPSTE24 mutations and to the degree of enzyme activity reduction. The total absence of ZMPSTE24 results in the higher amount of prelamin A and to the most severe phenotype, the lethal neonatal Restrictive Dermopathy (RD) [7]. Other mutations allowing a residual activity are associated with less severe clinical phenotypes such as Mandibulo-Acral Dysplasia (MAD) [8]. Here we report detailed clinical phenotype and cellular investigations for a patient carrying a heterozygous missense mutation in ZMPSTE24 and referred for common central obesity with metabolic syndrome (MS). This patient was reported initially in a cohort of several patients with severe metabolic syndrome and cell nuclei abnormalities, but with limited information provided about the clinical data and cellular senescence rate [9]. The causative mutation, p.L438F, was shown to reduce ZMPSTE24 activity drastically and produce an impaired capacity to process prelamin A maturation both *in vitro* [10] and in the patient’s cells, where the normal allele insufficiently compensates ZMPSTE24 deficiency [9]. In the present study, we show that fibroblasts carrying the mutation p.L438F exhibit accelerated senescence linked to prelamin A accumulation.

## 2. Materials and Methods

### 2.1. Cell Cultures

Primary fibroblast cultures were performed after patient’s skin biopsies. Control fibroblasts from a non-obese non-diabetic individual (strain 7095) were provided by the Centre de Ressources Biologiques Timone. Fibroblasts were cultivated in DMEM medium (Biowest, Nuaillé, France) supplemented with 15% fetal bovine serum (Eurobio, Courtaboeuf, France), 1% l-glutamine 200 mM (Life Technologies, Thermo Fisher Technologies) and 1% Penicillin-Streptomycin-Amphotericin (PAA Laboratory), in a culture flask of 25 cm^2^ (SPL Life Sciences, Korea), under controlled atmospheric conditions (10% O_2_, 5% CO_2_, and 85% N_2_) at 37 °C with 95% humidity.

### 2.2. Molecular Studies

DNA was extracted from patient’s fibroblasts using NucleoSpin^®^ Tissue XS (Machinery-Nagel, Germany) and purified using a QIAamp^®^ DNA Micro Kit (Quiagen, Germany) following the manufacturers protocols. The target regions corresponded to the following genes: SREBF1, RETN, PPARG, PLIN1, LEP, LEPR, GHRL, FKRP, BSCL2, ADIPOQ, AGPAT2, and FTO. The capture was performed with reagents from a custom design HaloPlex Target Enrichment kit (Agilent Technologies, Santa Clara, CA, USA), according to the HaloPlex Target Enrichment For Ion Torrent Sequencing Version D4. Libraries were quantified and qualified using the Qubit Fluorometer (Thermo Fisher Scientific Inc., USA) and the Agilent 2100 Bioanalyzer instrument (High Sensitivity DNA Kit) to enable equi-molar pooling of barcoded samples. Template preparation, emulsion PCR, and Ion Sphere Particles (ISP) enrichment were carried out using the Ion PI™ Template OT2 200 Kit v2 on the Ion OneTouch™ 2 System (Thermo Fisher Scientific Inc.). The quality of the ISPs was assessed using a Qubit 2.0 Fluorometer, and the ISPs were loaded and sequenced on a Ion PI™ Chip Kit v2 using Ion PI™ Sequencing 200 Kit v2 on the Ion Proton™ Sequencer (Thermo Fisher Scientific Inc.). Raw data were first aligned with the provided software suite included with the Ion Proton system to generate BAM files. The coverage and sequencing depth analysis were computed using the BEDtools suite v2.17 [11] and in-house scripts. Variants were identified using the Torrent Browser Variant caller (version 4.0.2), annotated and prioritized with the in-house “VarAFT” system that includes Annovar [12].

### 2.3. Immunofluorescence

Fibroblasts were grown on coverslips (Lab-tek, SPL Life Sciences) after 30 nM small interference RNA (siRNA) treatment for 48 h and fixed in 4% PAF solution (paraformaldehyde) for 10 min. The antibody used against lamin A/C (SC 6215, Santa Cruz Biotechnology Inc.) was associated with a secondary antibody coupled to Texas Red dye (donkey anti-goat IgG H & L, Alexa Fluor^®^ 594, ab150132, Abcam^®^, Paris, France). The fibroblast nuclei were stained by DAPI (diaminido-2phenylindole hydrochloride). Criteria for nuclear anomalies were: aberrant nuclear lamin A staining pattern, aberrant lamin A cytoplasmic localization, and aberrant nucleus shape. Between 100 and 150 cell nuclei were examined in each condition. Cells were observed at a ×100 objective and representative pictures of nuclear anomalies were taken on ApoTome (ZEISS, Germany) and worked with Image J (National Institutes of Health, USA).

### 2.4. Population Doubling Level

The population doubling level (PDL) was calculated with the mathematical relation log2 (D/D0) with Do representing the seeded cells and D the harvested cells [13]. The PDL was measured for four weeks (passages 10 to 21). Senescence was complete when PDL was inferior or equal to zero.

### 2.5. SiRNA

SiRNA treatment was directed against lamin A 3′UTR and a scrambled siRNA was used as a negative control. 40,000 and 150,000 fibroblasts were seeded in coverslips (Lab-Tek, SPL Life Science) and 6-well plates (VWR Plates, Fontenay-sous-Bois, France) for immunofluorescence and western blot analysis. When 70% confluent was reached, the fibroblasts were transfected using a JetPrime kit (Polyplus Transfection, Illkirch, France) with siRNA at 30 nM. The cells were then incubated for 48 h.

### 2.6. Western Blotting

Total fibroblast proteins were extracted in 200 μL of NP40 Cell Lysis buffer (Invitrogen, Carlsbad, CA, USA) with Protease and Phosphatase Inhibitor Cocktail (Thermo Scientific). Fibroblasts were incubated for 30 min at 4 °C, sonicated four times (30 s on/off each), and centrifuged at 10,000 *g* for 10 min. Protein concentration was quantified using a Pierce BCA Protein Assay Kit (bicinchoninic acid) and absorbance was measured at 562 nm using a Nanodrop 1000 (Thermo Fisher Scientific Inc.). 40 μg were loaded into 7% Tricine acetate gel (CriterionTM XT precast gel) using XT Tricine running Buffer (Biorad, USA). After electrophoresis, gels were electro transferred onto nitrocellulose membranes or Immobilon-FL polyvinylidene fluoride membranes (Millipore), blocked in Odyssey Blocking Buffer diluted 1:1 in TBS for 1 h at room temperature, and incubated overnight at 4 °C with primary antibodies. These primary antibodies were diluted in blocking buffer with 0.1% Tween 20, 1:1000 rabbit polyclonal anti-lamin A/C (sc-20681, Santa Cruz Biotechnology) and 1:40,000 monoclonal anti-glyceraldehyde-3-phosphate dehydrogenase (GAPDH) (MAB374, Merck Millipore). Membranes were washed with TBS and 0.1% Tween 20 and then incubated with 1:10,000 IR-Dye 800-conjugated secondary donkey anti-rabbit or IR-Dye 700-conjugated secondary anti-mouse antibodies (LI-COR Biosciences) in blocking buffer with 0.1% Tween 20 and 0.1% SDS (LI-COR Biosciences). Odyssey Infrared Imaging System (LI-COR Biosciences) was used to reveal the membranes. GAPDH was used as total cellular protein loading control.

### 2.7. Senescence-Associated Beta-Galactosidase Assay

Senescence-associated beta galactosidase activity was evaluated using the Beta-Glo^®^ Assay System (Promega Corporation, USA). The Beta-Glo^®^ reagent is constituted by 6-*O*-β-galactopyranosyl-luciferin substrate cleaved by β-galactosidase to form luciferin that is then catalyzed by luciferase in the presence of cofactors to produce light. The luminescent signal generated is proportional to the amount of β-galactosidase. Experiments were performed according to the manufacturer’s instructions, in 96-well white-walled plates (VWR, International SAS, Strasbourg, France). A volume of 100 μL of culture containing 10,000 cells was dropped into each well in quadruple. After 48 h, the culture medium was changed using DMEM without red phenol (Gibco^®^, Cell Culture, Invitrogen, Corporation, San Diego, CA, USA) supplemented with 10% fetal bovine serum (Eurobio, Courtaboeuf, France), 1% l-glutamine 20 mM (Life Technologies, Thermo Fisher Technologies) and 1% Penicillin-Streptomycin-Amphotericin (PAA Laboratory), to avoid interferences that can affect the luminescent signal. This was followed by the addition of 100 μL of Beta-Glo^®^ reagent per well 30 min before the measurement of the luminescence on the Glomax^®^ luminometer (Promega Corporation). Another experiment has been performed on cells cultured in the presence of 30 nM of siRNA targeting lamin A 3ʹUTR for 24 h, before the addition of Beta-Glo^®^ reagent. The experiments were performed five times on quadruple samples of control and patient at passage 16.

### 2.8. Cellular BrdU Labelling

Cell proliferation was assessed by 5-bromo-2′-deoxyuridine (BrdU) incorporation using the Cell proliferation ELISA, BrdU (colorimetric) Kit (Roche Applied Science). 10,000 fibroblasts were deposited in each well in a final volume of 100 μL and incubated for 24 h in humidified atmosphere at 37 °C. Then, 10 μL of BrdU was added to each well and cells were re-incubated for another 24 h period. The manufacturer’s protocol was followed and 5 min after the addition of the substrate, 25 μL of H_2_SO_4_ 1 M were added to each well allowing the reading of the absorbance at 450 nm (reference wavelength: 600 nm) on the Glomax^®^ reader (Promega Corporation). The experiments were performed five times on quadruples samples at passage 16.

### 2.9. Statistical Analysis

The sample size being too small to pass the Normality test (*n* = 5), we used the Mann-Whitney non-parametric test to assess statistical significance between the two groups (control and patient). An ANOVA test was used for the statistical analysis of PDL. The significance threshold was defined as *p* < 0.05. Statistical analyses and graphical representations were performed using GraphPad Prism 6.07 (GraphPad Software, San Diego, CA, USA).

## 3. Results

### 3.1. Patient Description

**Figure 1:**
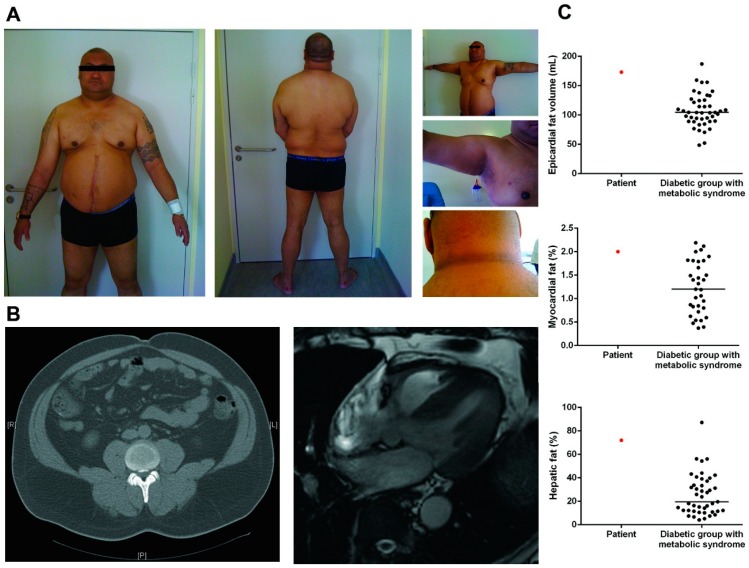
Patient’s description. (**A**) Photographs of the patient carrying the heterozygous *ZMPSTE24* missense mutation L438F, showing an accumulation of facial and cervical fat, android obesity, acanthosis nigricans, and no lower limb lipoatrophy; (**B**) Abdominal CT scan and cardiac-MRI of the patient showing the presence of superficial and deep subcutaneous adipose tissue and accumulation of cardiac ectopic fat; (**C**) Quantification of patient epicardial fat volume, myocardial, and hepatic triglyceride content using 3T MRI and proton magnetic resonance spectroscopy (^1^H-MRS). Comparison of patient’s ectopic fat depots with a group of type 2 diabetic subjects with metabolic syndrome matched for age and BMI.

The patient is a native of New Caledonia, a French overseas territory located in the Southwest Pacific Ocean. He was referred for android obesity associated with type 2 diabetes diagnosed at age 39 (weight at diagnosis > 100 kg), requiring insulin five years after initial diagnosis. At age 47, his height was 195 cm, weight = 144 kg, BMI = 37.9 kg/m^2^, waist circumference = 130 cm, hip circumference = 120 cm, thigh circumference = 63 cm. There were clinical and biological evidences of insulin resistance with acanthosis nigricans in the axillae and cervical regions (Figure 1A), increased fasting plasma insulin (89.4 mUI/L (normal range: 6.5–29.1) and homeostasis model assessment of insulin resistance (HOMA-IR at 29). However, his insulin requirement dose was relatively small (0.13 UI/kg), and he was only treated with basal insulin and oral antidiabetic drugs, with a good glycemic control (HbA1c = 7.5%). Physical examination revealed an imposing physical appearance, with android obesity and fat accumulation in the neck and face, supraclavicular filling, buffalo neck but no obvious clinical subcutaneous lipoatrophy, nor calf hypertrophy or muscle weakness (Figure 1A).

He suffered from hypertension, hypertriglyceridemia (fasting triglycerides = 2.05 g/L, HDL cholesterol = 0.38 g/L, LDL cholesterol = 0.46 g/L and total cholesterol = 1.25 g/L), was a current smoker, and reported current alcohol consumption. Routine investigations showed a slight increase in the level of Creatine Kinase (281 UI/L; normal range <170) without muscular or articular pain. Liver enzymes were in the normal range (AST 36 UI/L; ALT 35 UI/L; GGT 27 UI/L and PAL 84 UI/L). An abdominal echography showed hepatomegaly with hepatic steatosis (Figure 1B). Cardiac investigations revealed a dilated cardiomyopathy with a left ventricular ejection fraction (LV-EF) of 25%–30%, which required treatment with beta-blockers, diuretics, and angiotensin converting enzyme inhibitors. The 24 h ECG showed slight conduction disturbances with isolated polymorphic ventricular extrasystoles. After six years of treatment, the patient recovered normal echographic and Magnetic Resonance Imaging (MRI) LV-EF (50%). Pulmonary function testing showed a mild restrictive defect (−20%) and hypoxemia at rest (71 mmHg). An abdominal computed tomography (CT) scan confirmed that abdominal subcutaneous lipoatrophy was absent (Figure 1B).

The patient fulfilled all the International Diabetes Federation (IDF) diagnosis criteria for metabolic syndrome and also fulfilled all the criteria of National Cholesterol Education Program’s Adult Treatment Panel III (NCEP-ATP-III) definition with five criteria fulfilled out of five.

Serum adipokines were assessed and revealed that adiponectin was low 1 μg/mL (3.8–11.8), confirming the decrease in insulin sensitivity, and serum leptin was measured at 49.8 ng/mL (1–12.5).

Cardiovascular magnetic resonance imaging at 3T and proton magnetic resonance spectroscopy revealed increased ectopic fat accumulation (Figure 1B). In particular, epicardial fat volume was highly increased at 173 cm^3^, myocardial and hepatic triglycerides content were extremely high at 2% and 72%, respectively and were higher than in a group of type 2 diabetics with MS, matched for age and BMI (Figure 1C). The patient had a brother who was described as having the same morphotype, but familial genetic screening could not be performed as the patient had broken off their relationship.

Direct sequencing of *LMNA* and *ZMPSTE24* revealed the heterozygous missense mutation c. 1312C > T (p.L438F) in *ZMPSTE24* as the only variation in these genes. This mutation was predicted as pathogenic in silico (with a maximal score of 1 in Polyphen). To rule out the presence of another variant in another gene, we performed high throughput sequencing of a genes panel including those commonly associated with obesity, lipodystrophy, and metabolic syndrome. No other variant with non-ambiguous signification was evidenced.

### 3.2. Nuclear Shape Anomalies and Senescence Studies

**Figure 2:**
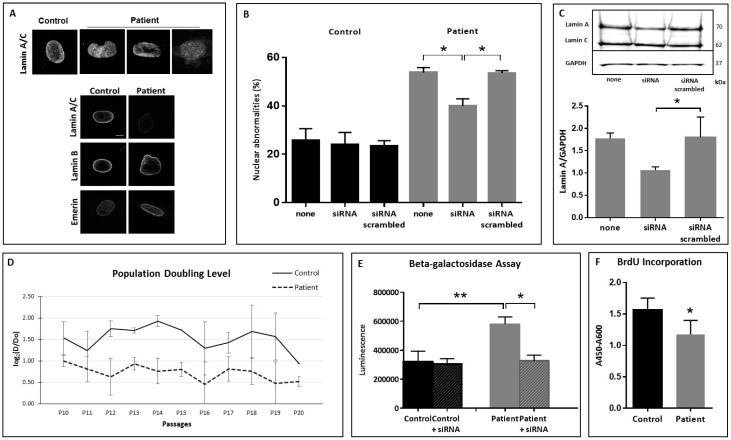
Nuclear shape anomalies and senescence tests. (**A**) Representative pictures of nuclear anomalies. Upper panel: aberrant nuclear pattern, aberrant cytoplasmic localization and aberrant shape after lamin A staining. Fibroblasts were examined with Apotome.2 (Zeiss) stereomicroscope under oil immersion (100× magnification). Lower panel: representative pictures of nuclear anomalies with different staining (Lamin A/C, Lamin B and Emerin); (**B**) Quantitative estimation of dysmorphic nuclei in control and patient fibroblasts with and without siRNA. Asterisks correspond to *p* values of 0.0268; (**C**) Representative Western Blot and quantitative estimation of lamin A in patient fibroblasts with and without siRNA. Asterisk corresponds to a *p* value of 0.0286; (**D**) Population Doubling Level. ANOVA showed *p* < 0.0001 between control and patient PDL; (**E**) Senescence related beta-galactosidase activity before and 24h after siRNA treatment. The double asterisk corresponds to a *p* value of 0.0079; the asterisk corresponds to a *p* value of 0.0159. (**F**) BrdU incorporation. Asterisk corresponds to a *p* value of 0.0173. In all experiments, “control” was fibroblasts from healthy people.

Altered lamin A staining was observed in patients cells with reduced signal and heterogeneous staining with aggregates whereas lamin B and Emerin staining were normal (Figure 2A). Nuclear shape anomalies were quantified in primary fibroblasts and in lymphoblastoïd cells at different passages and were found significantly increased when compared to control cells (Figure 2B).

In order to confirm that these nuclear anomalies were related to prelamin A accumulation, cells were cultured in the presence of siRNA directed specifically to the 3′ region of lamin A transcripts and aimed at reducing the translation of prelamin A. Decreased lamin A production following treatment by oligonucleotides was confirmed by Western Blotting and was estimated between 40% and 30% (Figure 2C). Then, abnormal nuclear shapes and lamin A staining were evaluated after siRNA treatment and showed that decreased lamin A production was associated to a significantly decreased number of abnormal nuclei in the patient cells (*p* = 0.0268) (Figure 2B).

Cellular senescence was investigated in primary fibroblasts by different approaches. Population Doubling Level (PDL) was estimated for control and patients fibroblasts. Whereas the PDL of control fibroblasts remained stable from passage 10 up to passage 19, the proliferation rate of fibroblasts carrying the ZMPSTE24 mutation L438F was constantly lower than the control and decreased rapidly after passage 16 (*p* < 0.0001 with an ANOVA test) (Figure 2D). In addition, the ZMPSTE-mutated fibroblasts had a significantly increased senescence-associated β-galactosidase activity, as compared to control cells at the same passage whereas β-galactosidase activity returned to control level when fibroblasts were cultured in the presence of siRNA interfering with the translation of prelamin A (Figure 2E). At last, fibroblasts displayed a striking decrease in replicative capacity, measured in terms of bromodeoxyuridine (BrdU) incorporation (Figure 2F). Altogether, senescence investigation showed a significantly increased senescence rate for the patient’s cells compared to control cells at the same passage (0.0001 < *p* < 0.0286 according to the experiments).

## 4. Discussion

In the present study, we describe the detailed phenotype of partial lipodystrophy with senescence features associated with a heterozygous missense mutation in ZMPSTE24 and prelamin A accumulation. Up to now, pathological phenotypes related to mutations in ZMPSTE24 were linked to homozygous or compound heterozygous mutations and resulted in RD, MAD, or progeria-like syndromes. Thus, the genetics of the ZMPSTE24 diseases would indicate that usually, haplo-insufficiency is not causative of a pathological phenotype. In order to confirm this point, we interviewed four unrelated individuals carrying the recurrent null mutation c. 1085_1086insT in heterozygous condition. These individuals were searched for ZMPSTE24 mutations since they have had a child with RD. All of them were more than 40 years old and one more than 60. None of them reported lipodystrophy, type 2 diabetes, or hypertriglyceridemia for them or for their close relatives (parents and siblings). This suggests strongly that the mutation L438F has a dominant negative effect leading to an insufficient compensation of decreased activity by the normal allele.

Familial partial lipodystrophies (FPL) are rare genetic diseases that share some features with the metabolic syndrome, making clinical diagnosis challenging. Central obesity with metabolic disturbances such as hypertension, insulin-resistance, or glucose intolerance is very common worldwide with the increase in the obesity epidemic [14]. Several studies have indicated that FPL may have a greater prevalence than previously thought due to a lack of recognition of subtle forms, especially when the extent of fat loss is moderate [15]. Decaudain *et al.* sequenced the *LMNA* coding regions in 277 unrelated adults investigated for lipodystrophy and/or insulin resistance and demonstrated that patients with non-codon 482-associated mutations often lacked lipodystrophy on physical examination [16]. A study of 5000 non-obese type 2 diabetic patients found that among those with a body mass index <27 kg/m^2^ and high insulin requirements (>100 U/day), there were five subjects with previously unrecognized FPL [17]. Accordingly, we identified in 87 patients with common MS, 11% with abnormal nuclear shape and disturbed lamin A/C nuclear distribution [9]. These studies strongly suggest that there is a high variability in clinical presentation for the partial lipodystrophies, and that FPL can be easily mistaken for common forms of abdominal obesity and MS [18]. In our case, the patient had a high BMI, with high serum leptin, and not so pronounced insulin-resistance in terms of insulin units per kg of body weight per day requirement. However, he had high HOMA-IR, low adiponectin levels, required insulin early in his disease natural history and suffered from dilated cardiomyopathy. Patients with lipodystrophies present metabolic consequences that are remarkably similar to those observed in obesity, but generally more severe, showing that maintaining a healthy subcutaneous amount of fat is an essential requirement for metabolic homeostasis [19]. Indeed, adipose tissue (AT) is critical in regulating lipid and glucose homeostasis. It buffers lipid excess from over-nutrition by sequestering free fatty acids (FFA) into triglycerides, thereby protecting the other organs from lipotoxicity [20]. In case of absent or dysfunctional subcutaneous AT, the neutral lipid storage capacity of the AT is exceeded and the flow of FFA is redirected to peripheral tissues, such as the muscle, the liver, and the heart. Severe ectopic fat accumulation is an important feature associated with FPL, which can lead to severe complications as the genetically limited expandability of AT drives the accumulation of lipids to non-adipose cells. A human non-invasive quantification of ectopic fat has been developed in recent years and could help in detecting FPL [21]. One recent study has confirmed in six patients with a mutation in *LMNA* that epicardial fat was significantly higher than in healthy controls with matching for sex, age, and BMI [22]. We also report here an extremely high myocardial triglyceride content in this case compared to type 2 diabetic subjects with matching age and BMI. Whether the increase in myocardial fat can be implicated in the occurrence of the dilated cardiomyopathy has been suggested [23,24] but merits further evaluation.

At the molecular level, FPL results from genetic defects either in fat differentiation and formation or from accelerated cellular senescence [25]. The pathophysiological link between partial lipodystrophy and cellular senescence has been previously evoked not only for *LMNA* mutations but also with the description of lipodystrophy syndrome resulting from mutations in WRN which encodes the DNA helicase WRN, involved in DNA replication and repair [26].

In the absence of familial study, the link between mutation and phenotype is not formally established in this patient. Nevertheless, we showed that the patient’s cells exhibit accelerated senescence and that the senescence rate is similar to the rate observed for fibroblasts carrying the R482W *LMNA* mutation, responsible for the FPL Dunnigan Type [27]. In cases of FPL linked to mutations in *LMNA*, the negative impact of causative mutations on the prelamin A processing rate has been well documented [28,29]. The mutated prelamin A that accumulates, binds SREBP1 at the nuclear rim, decreases the pool of active SREBP1 that normally activates PPARgamma, finally causing impairment of pre-adipocyte differentiation [30,31]. This mechanism is probably involved in the case we reported here, where prelamin A accumulates on account of decreased ZMPSTE24 activity. In addition, it has been shown recently that ZMPSTE24 downregulation is, in itself, a major contributor in Vascular Smooth Muscle Cell dysfunctions that could translate into early atherosclerosis at the clinical level [32].

## 5. Conclusions

We evidenced here at the cellular level, all the features of premature senescence observed in typical laminopathies: abnormal nuclear shape, decreased capacity to proliferate after several passages, decreased transcription activity as shown by BrdU incorporation, and increased senescence-related beta-galactosidase activity.

The patient described here was referred initially for metabolic syndrome complicated by cardiomyopathy and no limb lipoatrophy nor limb muscular hypertrophy. He presented several risk factors such as diabetes, hypertension, low HDL cholesterol, and smoking. This phenotype, although typical, is milder than those usually reported with FPLD type 2 or 3. Alcohol consumption may be a confounding factor and could have contributed to liver steatosis and to dilated cardiomyopathy. Penetrance may be incomplete and other risk factors may be necessary to reveal the clinical effects of premature senescence. For these reasons, this condition may be under-diagnosed among metabolic syndromes.
